# Correlates of Zooplankton Beta Diversity in Tropical Lake Systems

**DOI:** 10.1371/journal.pone.0109581

**Published:** 2014-10-16

**Authors:** Paloma M. Lopes, Luis M. Bini, Steven A. J. Declerck, Vinicius F. Farjalla, Ludgero C. G. Vieira, Claudia C. Bonecker, Fabio A. Lansac-Toha, Francisco A. Esteves, Reinaldo L. Bozelli

**Affiliations:** 1 Laboratório de Limnologia, Departamento de Ecologia, Instituto de Biologia, Universidade Federal do Rio de Janeiro, CCS, Cidade Universitária, Rio de Janeiro, RJ, Brazil; 2 Departamento de Ecologia, Instituto de Ciências Biológicas, Universidade Federal de Goiás, Goiânia, GO, Brazil; 3 Netherlands Institute of Ecology (NIOO-KNAW), Department of Aquatic Ecology, Wageningen, The Netherlands; 4 Faculdade UnB Planaltina, Universidade de Brasília, Área Universitária n. 1 - Vila Nossa Senhora de Fátima, Planaltina, Distrito Federal, Brazil; 5 Núcleo de Pesquisas em Limnologia, Ictiologia e Aqüicultura (NUPELIA), Universidade Estadual de Maringá, Jd. Universitário, Maringá, Paraná, Brazil; 6 Núcleo de Pesquisas em Ecologia e Desenvolvimento Sócio Ambiental de Macaé, Rodovia Amaral Peixoto, Macaé, RJ, Brazil; McGill University, Canada

## Abstract

The changes in species composition between habitat patches (beta diversity) are likely related to a number of factors, including environmental heterogeneity, connectivity, disturbance and productivity. Here, we used data from aquatic environments in five Brazilian regions over two years and two seasons (rainy and dry seasons or high and low water level periods in floodplain lakes) in each year to test hypotheses underlying zooplankton beta diversity variation. The regions present different levels of hydrological connectivity, where three regions present lakes that are permanent and connected with the main river, while the water bodies of the other two regions consist of permanent lakes and temporary ponds, with no hydrological connections between them. We tested for relationships between zooplankton beta diversity and environmental heterogeneity, spatial extent, hydrological connectivity, seasonality, disturbance and productivity. Negative relationships were detected between zooplankton beta diversity and both hydrological connectivity and disturbance (periodic dry-outs). Hydrological connectivity is likely to affect beta diversity by facilitating dispersal between habitats. In addition, the harsh environmental filter imposed by disturbance selected for only a small portion of the species from the regional pool that were able to cope with periodic dry-outs (e.g., those with a high production of resting eggs). In summary, this study suggests that faunal exchange and disturbance play important roles in structuring local zooplankton communities.

## Introduction

Beta diversity, that is, the change in species composition between habitat patches, is directly related to local or alpha-diversity (i.e., number of species within a particular habitat) and to regional or gamma-diversity (i.e., diversity in the different habitats within a region) [1], [2]. Understanding the mechanisms underlying beta diversity is one of the main goals in community ecology and interest in this area has increased substantially in the last decade [3], [4], [5].

Both deterministic and stochastic processes may affect beta diversity patterns. Deterministic processes are based on niche theory and assume that environmental filtering and biotic interactions play major roles in shaping local community composition. According to this theory, species have different ecological requirements, determining different responses to environmental gradients [6]. Stochastic processes are more related to the importance of colonization rates, random extinction and disturbance [7]. It is increasingly recognised that these processes shape the community structure simultaneously [8].

A number of factors have been shown to be important in predicting beta diversity. High spatial variation in environmental conditions allows species with different ecological requirements to occur in different sites, increasing beta diversity [9], [10], [11], [12]. Another important driver of beta diversity is the degree of spatial connectivity [10], [13], [14], [15]. Communities that are highly connected (e.g., by hydrological connections and smaller distance between habitats) may have lower beta diversity due to the higher exchange of individuals between these communities via active and passive dispersal. In addition, when connectivity is high, beta diversity can also decrease due to environmental homogenization [16], [17], [18], [19]. The degree of connectivity within a region can also vary over time. Lakes in river-floodplain systems, for example, tend to be highly connected to each other and with the main river during flood periods, leading to more similar communities and environmental conditions [17]. In addition, disturbances are, in general, responsible for a decrease in beta diversity [14], [20], [21], [22], as they impose environmental filters that select only portions of the species from the regional pools that are disturbance-tolerant [14], [21], [22]. On the other hand, disturbance can increase beta diversity in more isolated ponds as a result of stochastic recolonization and priority effects [23]. Temporary ponds undergo drastic disturbances, as they periodically dry up completely [24]. Finally, a positive relationship between beta diversity and primary productivity is expected due to a greater contribution of stochastic processes relative to deterministic ones in high productivity-environments [25], [26]. In regions with high productivity, a greater number of species can coexist and the composition of communities is likely to depend on the colonization history, such as priority effects [25], [27].

In this study, we tested the effects of environmental heterogeneity, spatial extent, hydrological connectivity (isolated vs. floodplain lakes), seasonality (wet season or high water period vs. dry season or low water period), disturbance (periodic dry-outs) and productivity (mean chlorophyll-*a* concentration) on beta diversity of zooplankton communities in five geographic regions of Brazil. We predicted that (a) lake systems that are more heterogeneous in their environmental conditions would have higher beta diversity, (b) regions where the spatial extent is larger (i.e., larger distances between studied local communities) would have higher values of beta diversity, (c) connected environments would have lower beta diversity than isolated environments due to their greater similarity in environmental conditions and/or greater dispersal rates, (d) beta diversity would be higher during the low water period or dry season, when there is less connectivity between environments, (e) temporary environments would have lower beta diversity as they undergo disturbances (periodic dry-outs), selecting species tolerant to these extreme conditions and (f) regions with higher primary productivity would have a higher beta diversity.

## Materials and Methods

### Ethics Statement

Collecting permits were provided by the Instituto Chico Mendes de Conservação da Biodiversidade – ICMBio (Brazilian Ministry of Environment). None of the species collected are considered threatened.

### Study Area

This study was based on the analysis of data collected in aquatic environments of five regions in Brazil: 24 lakes in Trombetas River floodplain (Amazonian region, Northern Brazil), 20 lakes in the Upper Paraná River floodplain (Southern Brazil), 32 lakes in the Middle Araguaia River floodplain (Central Brazil), 21 coastal lakes and ponds located in the Restinga de Jurubatiba National Park (Macaé, Southeast Brazil) and 23 lakes and ponds in the Amazonian upland region (Carajás, mean altitude 710 m, Northern Brazil) (Figure S1). The Trombetas, Paraná and Araguaia floodplain lakes are permanent and connected with the main river (at least during the high water periods in the case of the Paraná River), while the water bodies of Macaé and Carajás regions consist of permanent lakes and temporary ponds, respectively, with no hydrological connections between them, except for small and sporadic connections during the rainy season. Ponds were considered temporary if they completely dried up at least once during our study period.

### Field Sampling

Each region was sampled in two wet and two dry seasons (or high and low water periods), except for Araguaia, which was sampled in one high and one low water period (see Table S1 for more details about the sampling schedule). For each lake, water samples were analyzed for total nitrogen (µmol L^−1^), total phosphorus (µmol L^−1^) and chlorophyll-*a* (µg L^−1^). In the field, we also measured pH, dissolved oxygen (mg L^−1^) and conductivity (µS cm^−1^). Details of the methods employed for the determination of these environmental variables are described in [28], [29], [30].

In Trombetas, Macaé and Carajás, samples of zooplankton were collected either by filtering 100 L of water (collected with a bucket in the case of shallow lakes; i.e. <1 m depth) through a 50 µm mesh plankton net or by directly taking vertical hauls with a 50 µm plankton net (for deep lakes; i.e. >1 m depth). In Paraná and Araguaia regions, zooplankton samples were obtained by pumping 600 L and 1000 L of water, respectively, over a 50 µm mesh plankton net. Samples were immediately fixed with 4% formaldehyde. In the laboratory, zooplankton individuals were identified to the lowest possible taxonomic unit. Triplicate aliquots (1 ml) of zooplankton samples were counted in either a Sedgewick-Rafter cell under a microscope (for rotifers and nauplii) or in open chambers under a stereomicroscope (for cladocerans and copepods). At least 100 individuals per group (rotifers, nauplii, cladocerans and copepods) were counted in each aliquot, but in most of samples, these numbers were exceeded. Entire samples (rather than aliquots) were analysed to identify rare species. We verified that most species were sampled within each region, as the cumulative richness curves tended to reach an asymptote (Figure S2).

### Data Analysis

We made an *a priori* distinction between three categories of lakes based on their degree of hydrological isolation and permanency. These categories consisted of permanent and connected (all lakes in Trombetas, Araguaia and Paraná), permanent isolated and temporary isolated lakes (the latter two groups being present only in Macaé and Carajás). For each category of lakes in each region, we separately calculated beta diversity for each of the sampling periods. Beta diversity was calculated as the mean distance of individual observations (e.g., lakes) to the group centroid (combination of lake category and sampling period), using a permutational analysis of multivariate dispersions (PERMDISP [31]). PERMDISP can be used for calculating beta diversity with the use of any dissimilarity measure [12], [31]. Because dissimilarity measures have different properties and can generate different beta diversity patterns, we applied PERMDISP to multiple distance measures, such as Bray-Curtis (DistC_BC_), 1-Jaccard (DistC_Jac_) and Simpson (DistC_Sim_) indices. Bray-Curtis is an abundance-based index, while Jaccard is based on presence/absence data. The pairwise Simpson dissimilarity coefficient [32] is also based on presence/absence data, but unlike Jaccard, it is independent of differences in local richness. Species abundance data were log (x+1) transformed prior to analysis.

Environmental heterogeneity and spatial extent were calculated by applying PERMDISP on Euclidean distances from standardized environmental variables (total nitrogen, total phosphorus, pH, dissolved oxygen and electrical conductivity) and geographic coordinates (decimal degrees), respectively. In all analyses, environmental heterogeneity and spatial extent were continuous variables, while connectivity, seasonality and disturbance were dummy variables with two levels (connected vs. isolated, high water vs. low water period, with (temporary) vs. without drought (permanent), respectively).

Linear mixed-effects models (LMMs) with restricted maximum likelihood estimation were used to examine the effects of connectivity (connected vs. isolated), seasonality (high water vs. low water period), environmental heterogeneity and spatial extent on each of the beta diversity measures (DistC_BC_, DistC_Jac_ and DistC_Sim_). In these analyses, region was specified as a random factor to account for non-independence of data. The effect of connectivity was evaluated only for permanent lakes because there were no connected temporary lakes in our dataset. Using data from the regions with both temporary and permanent lakes (Macaé and Carajás; *n = *16, 8 temporary and 8 permanent), we applied LMM to test for the effect of disturbance (periodic dry out) on beta diversity also incorporating seasonality, environmental heterogeneity and spatial extent as explanatory variables in the model. Connectivity, disturbance, season, spatial extent and region may affect beta diversity directly as well as indirectly through their effect on environmental heterogeneity. To obtain a better understanding of the potential importance of such indirect effects, we also performed LMMs to analyze the dependence of environmental heterogeneity on the other explanatory variables. Significance (*P*<0.05) was tested with using Type II tests (Wald *F* tests with Kenward-Roger degrees of freedom). Linear mixed-effects models were used to test the relationship between beta diversity measures and mean chlorophyll-*a* concentration (a proxy for productivity) and were performed separately because this variable was not available for all sampling times.

We also examined the effect of seasonality (high water vs. low water period) and disturbance (permanent vs. temporary lakes) on zooplankton communities by calculating the local contribution to beta diversity (LCBD) as a measure of ecological uniqueness of each lake in terms of species composition [33]. LCBS values “indicate the sites that contribute more (or less) than the mean to beta diversity” [33]. To test the effect of seasonality on zooplankton beta diversity of floodplain lakes (i.e., Trombetas, Paraná and Araguaia regions) we calculated LCBD for each region and for each year of sampling (i.e., data from one sampling during low and high water periods). According to our hypothesis, one would expect higher values of LCBD during the low water periods. Similarly, using data obtained in Carajás and Macaé regions from each sampling period, we calculated LCBD in order to test the effect of disturbance on beta diversity (especially, whether permanent lakes contribute more to the overall beta diversity than temporary lakes). All LCBD values were tested by permutation (999 runs) according to the procedures described in Legendre and De Cáceres [33].

All analyses were carried out in R 2.15.1 [34]. PERMDISP was performed using the *vegan* package [35], the pairwise Simpson dissimilarity coefficient was calculated using the *betapart* package [36], LMMs were fitted with the *lmer* function from the package *lme4* [37], Wald tests were carried out using the *car* package [38] and marginal R^2^ values (variance explained by fixed factors) [39] were calculated using the *MuMIn* package [40]. LCBD values were calculated using the functions provided by Legendre and De Cáceres [33].

## Results

We detected a total of 156 zooplankton species in Trombetas River floodplain, 208 in Paraná River floodplain, 128 in Araguaia River floodplain, and 168 and 120 species in the isolated lakes of Macaé and Carajás regions, respectively (for species lists and information on dominance, see Table S2). In general, species richness (according to a specific number of samples as, for instance, 40 samples, or to the mean values; see Figures S2 and S3, respectively) tended to be higher in lakes from the Paraná River floodplain and the Macaé region than in lakes from the other floodplains (Trombetas and Araguaia) and the Carajás region. Rotifers, when compared to other broad taxonomic groups, dominated the communities both in terms of species richness and abundance (except in the lakes from the Araguaia River floodplain, where cladocerans were more abundant; Figure S4).

**Figure 1:**
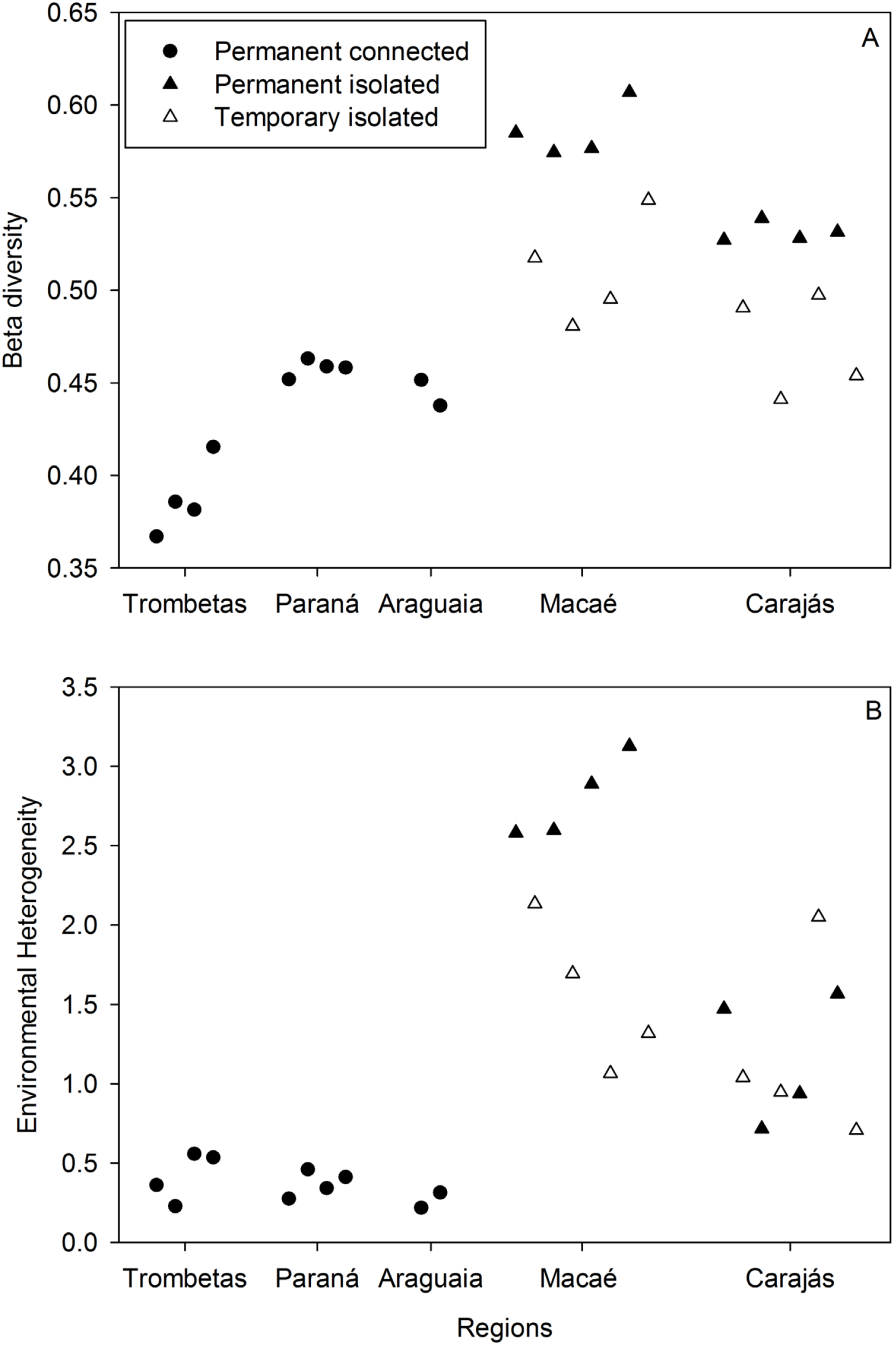
Zooplankton beta diversity for each studied region. (A) Zooplankton beta diversity (as the mean Jaccard distance to group centroid) for each region, sampling time (for each region, the different data points in the X-axis represent the different sampling times) and lake categories (permanent connected, permanent isolated and temporary isolated). (B) Environmental heterogeneity for each region, sampling time and lake categories.

Patterns of zooplankton beta diversity were similar independently of the community dissimilarity metric used (see Figures 1A and S5). For this reason, we only present results based on Jaccard distances. Beta diversity in regions with permanent lakes responded significantly to connectivity (Table 1; for models based on other dissimilarity coefficients, see Table S3), where regions with connected lakes showed lower values than regions with isolated lakes (Figure 1A). The lowest beta diversities were found in the lakes associated to the Trombetas River floodplain (Figure 1A), which has the highest connectivity level of all regions. Environmental heterogeneity, seasonality and spatial extent had no significant effect on beta diversity (Table 1).

**Table 1:** Summary of the linear mixed-effects model of zooplankton beta diversity measured as the mean1-Jaccard distance to group (DistC_Jac_) centroid for connectivity data (connected and isolated permanent lakes from all study regions).

Sample size: n = 18 observations						
Group: regions = 5						
Marginal R^2^ = 0.68						
DistC_Jac_	Random effect	Variance component				
	Region	0.00191				
	Residual	0.00018				
	**Fixed effects**	**Estimate**	**SE**	***df***	***t***	***P***
	Intercept	0.421	0.048		8.701	
	Connectivity (isolated)	0.119	0.051	1	2.344	0.047
	Environmental heterogeneity	0.007	0.012	1	0.638	0.571
	Spatial extent	0.010	0.110	1	0.096	0.932
	Seasonality (wet)	0.004	0.006	1	0.615	0.551

The intercept corresponds to expected beta diversity in connected lakes during the dry season, when environmental heterogeneity and spatial extent are zero. Marginal R^2^ represents the variance explained by fixed factors.

Beta diversity in the isolated lakes of Carajás and Macaé responded significantly to disturbance, but not to seasonality, environmental heterogeneity or spatial extent (Table 2; for models based on other dissimilarity coefficients, see Table S4). Within these regions, beta diversity was consistently higher in permanent than in temporary lakes (Figure 1A). The global models were similar to the reduced models in showing that only connectivity and disturbance were significant correlates of beta diversity (*t* = 3.72, *P* = 0.037 and *t* = −6.40, *P*<0.0001, respectively). The relationship between zooplankton beta diversity and the mean chlorophyll-*a* concentration was not statistically significant (*t* = −1.28, *P* = 0.22).

**Table 2:** Summary of the linear mixed-effects model of zooplankton beta diversity measured as the mean1-Jaccard distance to group (DistC_Jac_) centroid for disturbance data (permanent and temporary aquatic systems from Macaé and Carajás).

Sample size: n = 16 observations						
Group: regions = 2 (Macaé and Carajás)						
Marginal R^2^ = 0.78						
DistC_Jac_	Random effect	Variance component				
	Region	0.00007				
	Residual	0.00046				
	**Fixed effects**	**Estimate**	**SE**	***df***	***t***	***P***
	Intercept	0.626	0.071		8.846	
	Disturbance (temporary)	−0.060	0.012	1	−4.864	<0.0001
	Environmental heterogeneity	0.014	0.010	1	1.286	0.293
	Spatial extent	−0.720	0.419	1	−1.718	0.808
	Seasonality (wet)	0.003	0.011	1	0.258	0.719

The intercept corresponds to expected beta diversity in permanent lakes during the dry season, when environmental heterogeneity and spatial extent are zero. Marginal R^2^ represents the variance explained by fixed factors.

In agreement with the results of the LMMs, the LCBD values for lakes sampled during low and high water periods were similar (Figure S6), suggesting no effect of seasonality on zooplankton beta diversity in these regions. Moreover, the contribution of permanent lakes to zooplankton beta diversity in Macaé and Carajás regions was higher than the contribution of temporary ponds, with the latter showing lower and mostly non-significant values (Figure S7).

Environmental heterogeneity tended to be lower in connected than in isolated lakes (Figure 1B), but the difference was not statistically significant (*t* = 1.69, *P* = 0.08). Environmental heterogeneity was also unrelated to seasonality, spatial extent and disturbance (Table S5).

## Discussion

The hypothesis that beta diversity would be lower in hydrologically connected lakes was corroborated, independently of the measure of beta diversity utilized (i.e., Bray-Curtis, Jaccard or Simpson dissimilarity coefficients). Connectivity is known to increase the similarity in species composition in aquatic systems in two different ways. First, higher connectivity increases the similarity in environmental conditions among lakes [16], [17], [19]. Second, hydrological connectivity can also facilitate the exchange of organisms, via passive dispersal, among connected lakes, increasing the similarity in species composition [13], [14], [16], [41], [42], [43], even when environmental conditions are heterogeneous among lakes. The effects of environmental heterogeneity could be ruled out, as these effects were not significant, nor was there a significant relationship between environmental heterogeneity and connectivity. Hydrological connectivity therefore likely influences beta diversity primarily by increasing dispersal rates among the lakes, homogenizing zooplankton composition.

Our results also indicated that beta diversity was lower among temporary environments than among permanent ones within the same region. Environments that undergo disturbances, such as periodic dry-outs, are considered extreme environments. Thus, a particular group of species can persist under these conditions, representing a strong environmental filter [14], [21], [22], [24], [44], [45], but see [46]. The species that are able to produce more resting eggs and can hatch more rapidly after refilling are more likely to be positively selected to occur in temporary ponds [24]. Indeed, some studies have shown that rotifers of temporary ponds are characterized by a higher production of resting eggs than rotifers from permanent lakes [47], [48], [49]. Moreover, an enclosure experiment (Lopes et al., in preparation) performed in the region of Macaé showed that most common and dominant species present in the studied temporary environments of this region are able to produce resting eggs that rapidly hatch after the ponds are refilled (e.g., rotifers *Cephalodella gibba*, *Lecane bulla, L. leontina, Lepadella patella*, cladocerans *Coronatella monacantha, Ephemeroporus barroisi, Diaphanosoma birgei, Ilyocryptus spinifer* and the calanoid copepod *Diaptomus azureus*). Some of them, in particular, rotifers, were also able to colonize the ponds rapidly by aerial dispersal (less than 20 days after refilling). Priority effects may also play a key role in structuring these communities. The sequence in which species are added to an environment can facilitate or inhibit the establishment of other species, thus affecting the composition of communities [23], [50], [51]. Species that first established in an environment are more likely to be competitively superior to those arriving later [52], [53]. This effect is even stronger in communities that have dense resting egg banks [23]. In short, although the majority of zooplankton produce resting eggs, some species produce more than others. Species also differ in the speed with which they respond to hatching cues and in the viability of the eggs. Thus, we infer that the communities of temporary ponds would be assembled mainly by species that simultaneously have higher production of resting eggs and respond more quickly to hatching cues. Finally, although we did not test the effect of hydroperiod on zooplankton beta diversity, it is important to point out that the degree of water permanency is likely to influence beta diversity. According to the intermediate disturbance hypothesis, species diversity is higher at intermediate levels of disturbance, assuming a unimodal relationship between diversity and disturbance [54]. On the other hand, the relationship between disturbance level and beta diversity is more likely to be negative, where temporary ponds have the most similar species composition (low beta diversity). Therefore, semi-permanent ponds are expected to have higher species richness and intermediate values of beta diversity in comparison to permanent and temporary ones [14]. Further studies should test these predictions (but see [55] for a strong criticism of the intermediate disturbance hypothesis).

According to niche theory, environmental factors and biotic interactions act as strong filters that select species that can persist within a community [56], [57], [58]. This implies that beta diversity should increase with environmental heterogeneity. Indeed, a relationship between beta diversity and environmental heterogeneity has been demonstrated by studies conducted at different spatial scales [59], [60], [61], [62], [63]. In contrast, we did not find an effect of environmental heterogeneity on zooplankton beta diversity. We cannot exclude the possibility that we may have missed some important environmental variables for zooplankton beta diversity. However, although environmental heterogeneity should intuitively have an influence on beta diversity, there are also other studies that failed to show a significant relationship between these variables (see [64] and references therein). Given the uncertainty on the relative importance of environmental heterogeneity in predicting beta diversity, we are of the opinion that this question is still open to further research.

According to the neutral theory of biodiversity [65], beta diversity should increase with increasing distance between the habitat patches exclusively due to dispersal limitation. There is also evidence, including in the systems we studied here (see [17], [43]), that floods increase environmental similarity among floodplain lakes within a region, decreasing beta diversity. However, neither spatial extent nor floods were significant correlates of beta diversity. The distance between aquatic environments may not be large enough to create effective barriers to dispersal and zooplankton dispersal can be effective enough to occur even during events of low hydrological connectivity (i.e., low water period or dry season) and/or species establishment in new habitats may be a consequence of their dispersal during periods of high connectivity. The degree of isolation of an aquatic environment is perceived differently by different groups of organisms and will depend, for example, on their dispersal abilities, which may be related to characteristics such as body size. Zooplankters are believed to be efficient passive (overland) dispersers, especially over small scales (see [66], [67]). Besides being small-bodied, most species have the ability to produce resting eggs, which increases the likelihood of dispersal by wind and animal vectors [66], [68]. However, dispersal abilities may vary between the zooplankton groups. For instance, while copepods reproduce only sexually, rotifers and cladocerans are parthenogenetic organisms, thus probably increasing their chances of establishment, especially in more isolated and temporary environments [69]. Furthermore, we showed that the presence of hydrological connections can highly increase dispersal rates between environments in relation to overland dispersal. The latter may still be effective but dispersal rates are higher between connected aquatic ecosystems (but see [70]).

We expected beta diversity to be higher in regions with higher productivity (i.e., higher mean chlorophyll-*a* concentration) due to the greater contribution of stochastic factors in relation to deterministic ones in regions with higher productivity [25], [27], [71]. However, this relationship was not observed in this study. The lack of relationship between beta diversity and mean chlorophyll-*a* (a proxy for productivity) cannot be explained by the lack of sufficient variation in chlorophyll-*a* concentrations, which was wide between the regions. Thus, further studies are needed to understand the role of productivity in determining zooplankton beta diversity.

In conclusion, we showed that zooplankton beta diversity in the five regions studied here is mainly associated with hydrological connectivity and disturbances caused by droughts. The hydrological connectivity in the studied areas may act by facilitating the exchange of species among habitats, whereas droughts impose a strong environmental filter that selects for species that can cope with this disturbance. A fruitful avenue for further research would be to extend the approach used here to test specific correlates of zooplankton beta diversity patterns in reservoirs (which are environments highly different from those analysed in our study). For instance, the datasets obtained by [72], [73] could be used to test the role of reservoir trophic status and hydrological variation in predicting zooplankton beta diversity.

## Supporting Information

Figure S1 **Map showing the location and number of lakes (** ***n*** **) sampled in each study region in Brazil.**
(TIF)

Figure S2 **Cumulative species richness curves for each study region.**
(TIF)

Figure S3 **Gamma and alpha diversities for each region.** (A) Zooplankton gamma diversity for each region, sampling time (for each region, the different data points in the X-axis represent the different sampling times) and lake categories (permanent connected, permanent isolated and temporary isolated). (B) Mean zooplankton alpha diversity (as the mean Simpson distance to group centroid) for each region, sampling time and lake categories. The error bars represent the standard errors of the mean over aquatic environments.
(TIF)

Figure S4 **Mean species richness and abundance for each zooplankton group.** (A) Mean number of species for each zooplankton group (Rotifera, Cladocera, Copepoda), region (Trom, Trombetas; Par, Paraná; Arag, Araguaia; Mac, Macaé; Car, Carajás) and lake category (P, permanent; T, temporary). (B) Mean abundance (ind/mL) for each group, region and lake category. The error bars represent the standard errors of the mean over aquatic environments.
(TIF)

Figure S5 **Zooplankton beta diversities (as the mean Bray-Curtis and Simpson distances to group centroid) for each region.** (A) Zooplankton beta diversity (as the mean Bray-Curtis distance to group centroid on biological data based on log_10_ transformation) for each region, sampling time (for each region, the different data points in the X-axis represent the different sampling times) and lake categories (permanent connected, permanent isolated and temporary isolated). (B) Zooplankton beta diversity (as the mean Simpson distance to group centroid) for each region, sampling time and lake categories.
(TIF)

Figure S6 **Local contribution to beta diversity (LCBD) for Trombetas, Paraná and Araguaia regions.** Maps of Trombetas, Paraná and Araguaia regions during high and low water periods showing the local contributions to beta diversity (LCBD) of the zooplankton community at the study lakes. Size of the circles is proportional to the LCBD. Lakes in red have significant LCDB indices (*P*<0.05). 1 = first sampling year, 2 = second sampling year.
(TIF)

Figure S7 **Local contribution to beta diversity (LCBD) for Macaé and Carajás regions.** Maps of Macaé and Carajás regions during dry and wet seasons showing the local contributions to beta diversity (LCBD) of the zooplankton community at the study lakes. Size of the circles is proportional to the LCBD. Lakes in red have significant LCDB indices (*P*<0.05). All lakes in red are permanent lakes, except for the lakes in Macaé 3 and Macaé 4 labelled with T (temporary). 1 = first sampling time, 2 = second sampling time, 3 = third sampling time, 4 = fourth sampling time.
(TIF)

Table S1 **Sampling schedule for each study region.** Type of environment, date of collection and number of sampled aquatic environments (*n*) in each region, season and sampling time. Con, connected; Isol, isolated; Perm, permanent; Temp, temporary.
(DOCX)

Table S2 **Species list for each region.** List of species per region and lake category. Dominant species are highlighted in yellow. Tr, Pr, Ar, Ma and Ca = Trombetas, Paraná, Araguaia, Macaé and Carajás, respectively. PC, TI, PI = permanent connected, temporary isolated, permanent isolated.
(DOCX)

Table S3 **Linear mixed-effects models of zooplankton beta diversity measured as the mean Bray-Curtis and Simpson distances to group centroid for connectivity data.** Summary of the linear mixed-effects models of zooplankton beta diversity measured as the mean Bray-Curtis (DistC_BC_) and Simpson (DistC_Sim_) distance to group centroid for connectivity data (connected and isolated permanent lakes from all study regions). Marginal R^2^ represents the variance explained by fixed factors.
(DOCX)

Table S4 **Linear mixed-effects models of zooplankton beta diversity measured as the mean Bray-Curtis and Simpson distances to group centroid for disturbance data.** Summary of the linear mixed-effects models of zooplankton beta diversity measured as the mean Bray Curtis (DistC_BC_) and Simpson (DistC_Sim_) distance to group centroid for disturbance data (permanent and temporary aquatic systems from Macaé and Carajás). Marginal R^2^ represents the variance explained by fixed factors.
(DOCX)

Table S5 **Linear mixed-effects models of environmental heterogeneity for connectivity and disturbance datasets.** Summary of the linear mixed-effects models of environmental heterogeneity (Env) measured as the mean Euclidean distance to group centroid for connectivity (connected and isolated permanent lakes from all studied regions) and disturbance datasets (permanent and temporary aquatic systems from Macaé and Carajás). Marginal R^2^ represents the variance explained by fixed factors.
(DOCX)
