# Supplementary figures and images for: Correlates of Zooplankton Beta Diversity in Tropical Lake Systems
Source: PLoS One. 2014 Oct 16;9(10):e109581. doi: 10.1371/journal.pone.0109581 (PMC4199600; doi:10.1371/journal.pone.0109581)

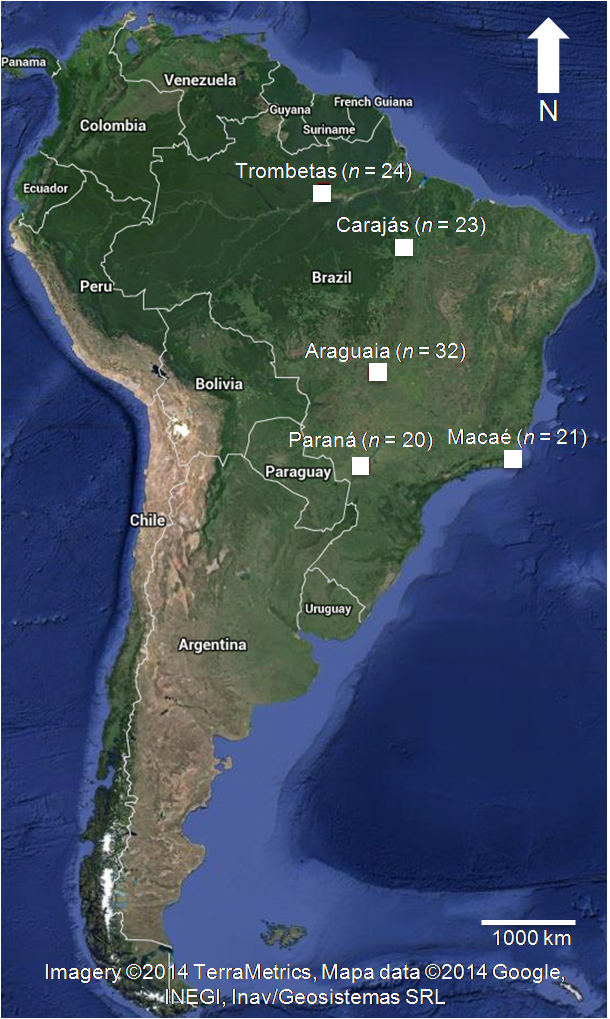

Supplement: Figure S1 — Map showing the location and number of lakes ( n ) sampled in each study region in Brazil. (TIF) [file pone.0109581.s001.tif]

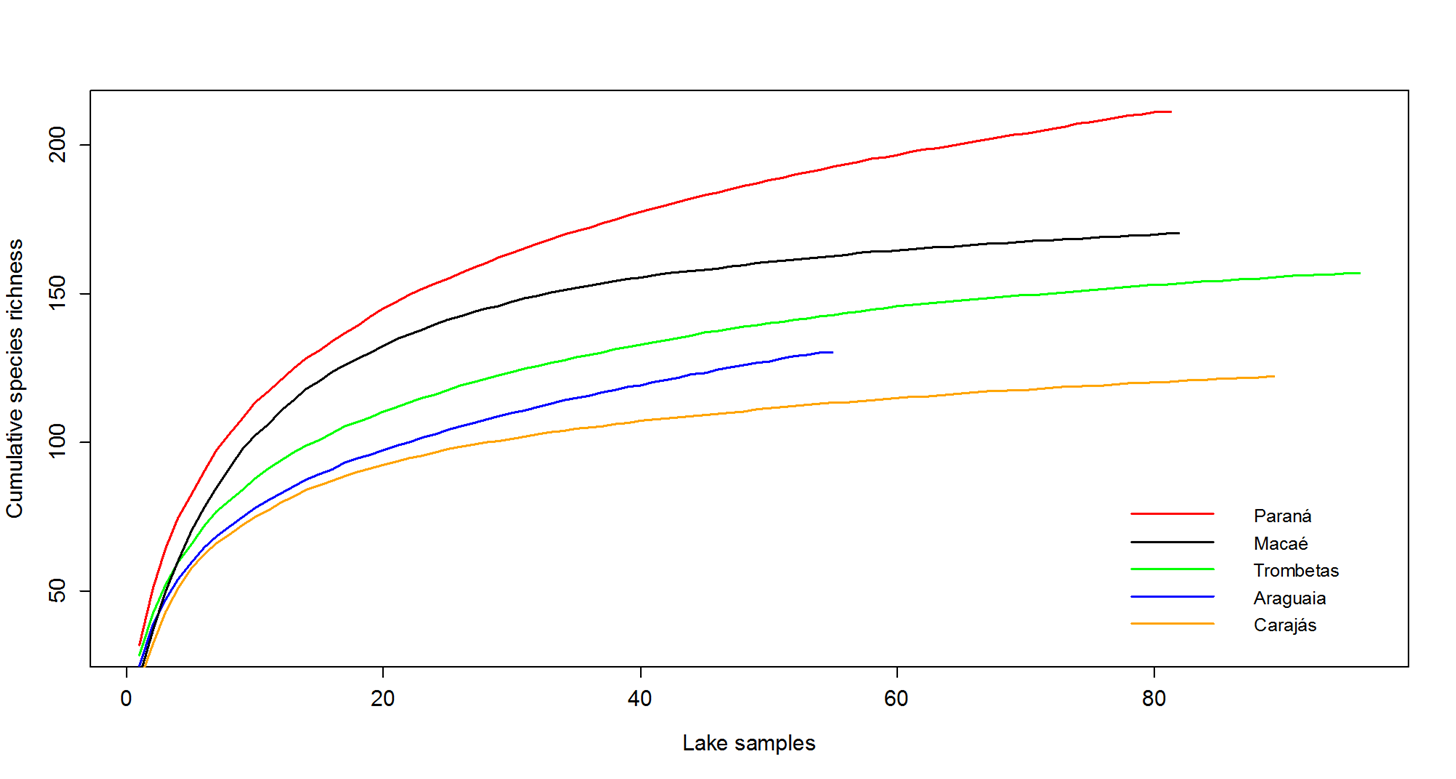

Supplement: Figure S2 — Cumulative species richness curves for each study region. (TIF) [file pone.0109581.s002.tif]

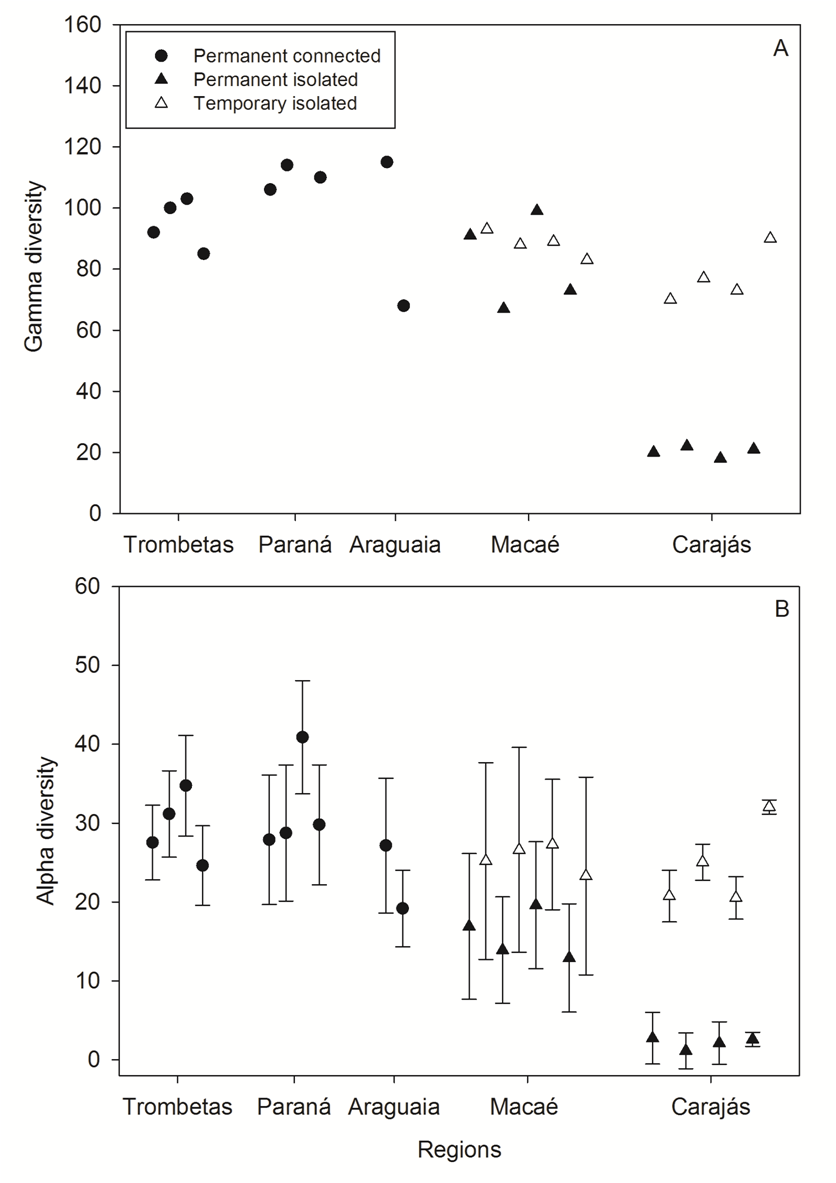

Supplement: Figure S3 — Gamma and alpha diversities for each region. (A) Zooplankton gamma diversity for each region, sampling time (for each region, the different data points in the X-axis represent the different sampling times) and lake categories (permanent connected, permanent isolated and temporary isolated). (B) Mean zooplankton alpha diversity (as the mean Simpson distance to group centroid) for each region, sampling time and lake categories. The error bars represent the standard errors of the mean over aquatic environments. (TIF) [file pone.0109581.s003.tif]

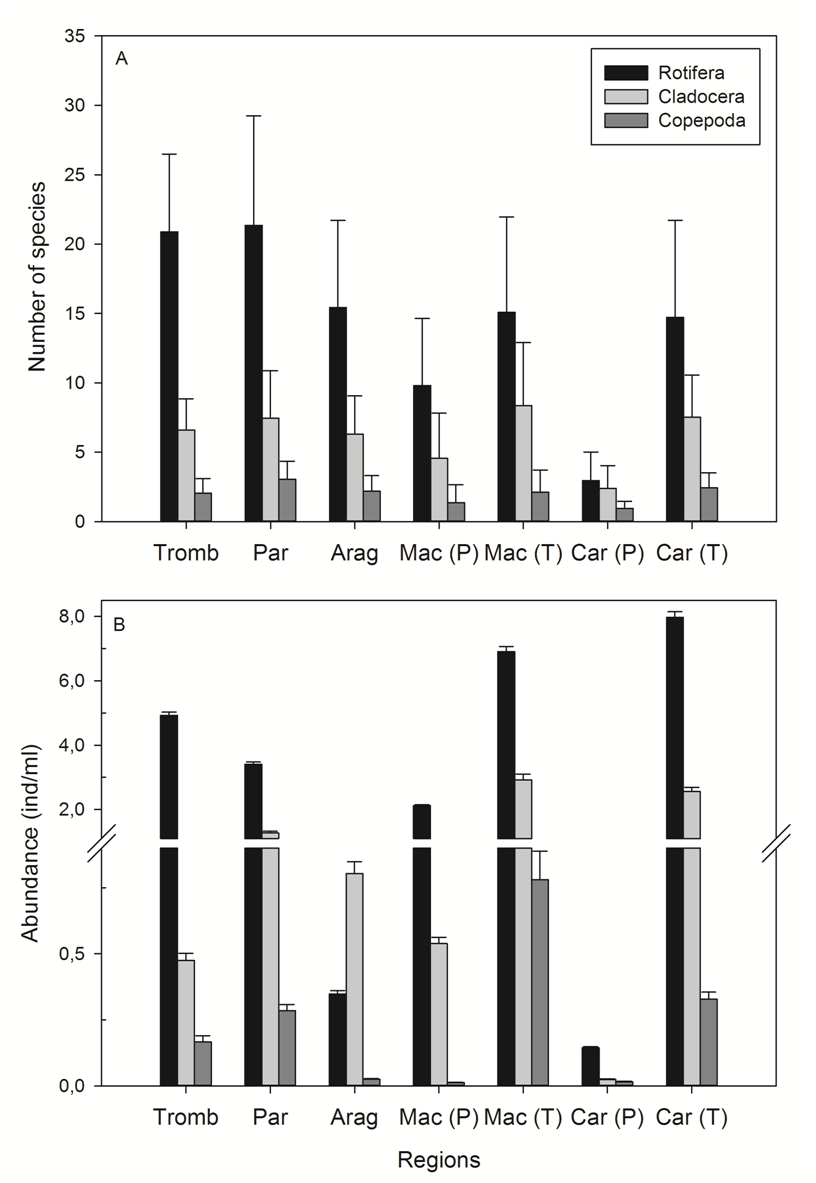

Supplement: Figure S4 — Mean species richness and abundance for each zooplankton group. (A) Mean number of species for each zooplankton group (Rotifera, Cladocera, Copepoda), region (Trom, Trombetas; Par, Paraná; Arag, Araguaia; Mac, Macaé; Car, Carajás) and lake category (P, permanent; T, temporary). (B) Mean abundance (ind/mL) for each group, region and lake category. The error bars represent the standard errors of the mean over aquatic environments. (TIF) [file pone.0109581.s004.tif]

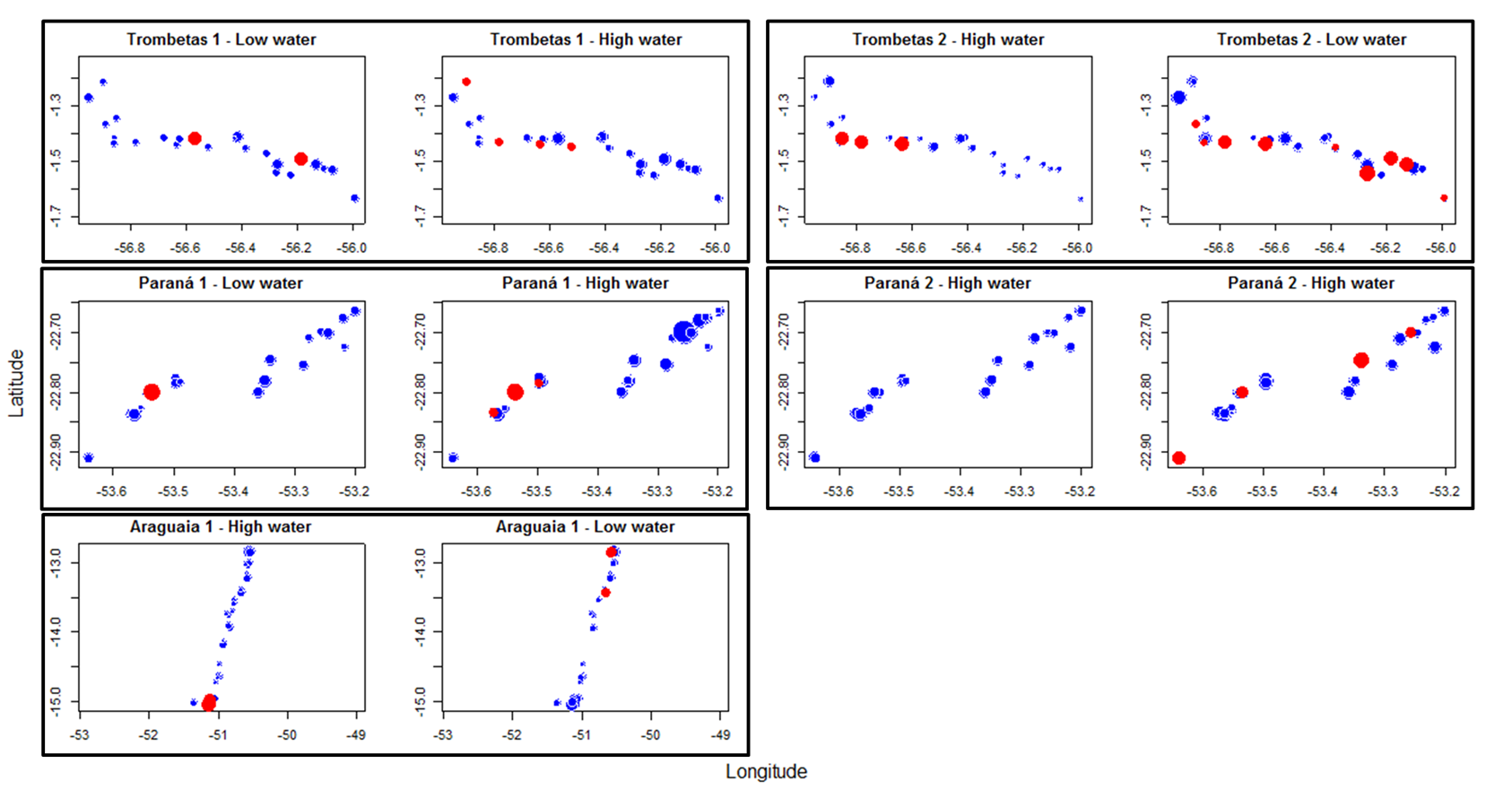

Supplement: Figure S6 — Local contribution to beta diversity (LCBD) for Trombetas, Paraná and Araguaia regions. Maps of Trombetas, Paraná and Araguaia regions during high and low water periods showing the local contributions to beta diversity (LCBD) of the zooplankton community at the study lakes. Size of the circles is proportional to the LCBD. Lakes in red have significant LCDB indices (P<0.05). 1 = first sampling year, 2 = second sampling year. (TIF) [file pone.0109581.s006.tif]

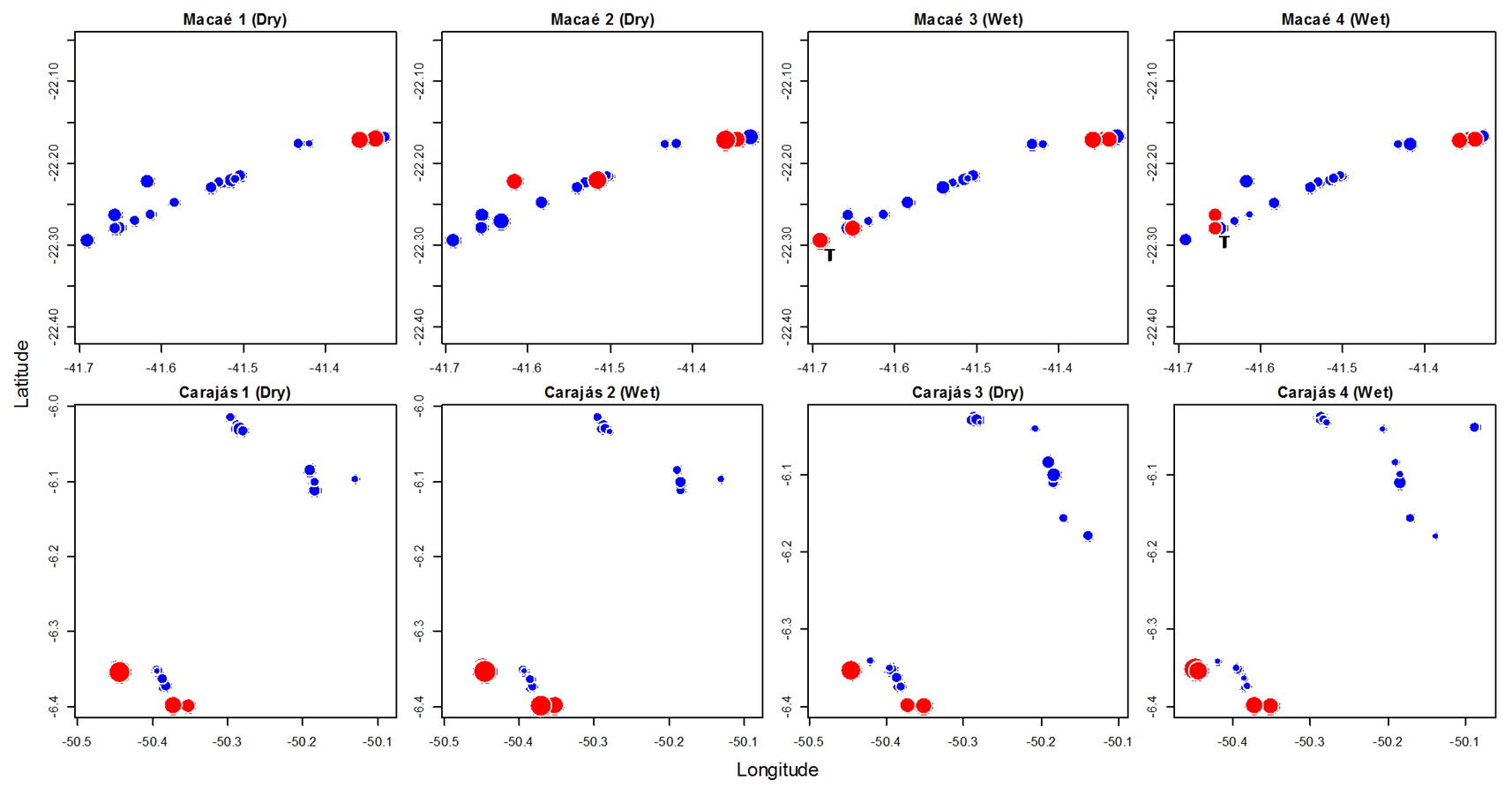

Supplement: Figure S7 — Local contribution to beta diversity (LCBD) for Macaé and Carajás regions. Maps of Macaé and Carajás regions during dry and wet seasons showing the local contributions to beta diversity (LCBD) of the zooplankton community at the study lakes. Size of the circles is proportional to the LCBD. Lakes in red have significant LCDB indices (P<0.05). All lakes in red are permanent lakes, except for the lakes in Macaé 3 and Macaé 4 labelled with T (temporary). 1 = first sampling time, 2 = second sampling time, 3 = third sampling time, 4 = fourth sampling time. (TIF) [file pone.0109581.s007.tif]
