# Supplementary material for: Correlates of Zooplankton Beta Diversity in Tropical Lake Systems
Source: PLoS One. 2014 Oct 16;9(10):e109581. doi: 10.1371/journal.pone.0109581 (PMC4199600; doi:10.1371/journal.pone.0109581)
Supplement: Table S1 — Sampling schedule for each study region. Type of environment, date of collection and number of sampled aquatic environments (n) in each region, season and sampling time. Con, connected; Isol, isolated; Perm, permanent; Temp, temporary. (DOCX) [file pone.0109581.s008.docx]

**Table S1. Sampling schedule for each study region.** Type of environment, date of collection and number of sampled aquatic environments (*n*) in each region, season and sampling time. Con, connected; Isol, isolated; Perm, permanent; Temp, temporary.

| **Region** | **Type** | **Wet seasons** | | | |  | **Dry seasons** | | | |
| --- | --- | --- | --- | --- | --- | --- | --- | --- | --- | --- |
|  |  | **1^st^ Sampling** | **n** | **2^nd^ Sampling** | **n** |  | **1^st^ Sampling** | **n** | **2^nd^ Sampling** | **n** |
| Trombetas | Con / Perm | Jun/08 | 24 | Jun/09 | 24 |  | Dec/07 | 24 | Jan/10 | 24 |
| Paraná | Con / Perm | Nov/00 | 20 | Mar/10 | 20 |  | May/00 | 20 | Set/10 | 20 |
| Araguaia | Con / Perm | Jan/06 | 32 |  |  |  | Jul/06 | 22 |  |  |
| Macaé | Isol / Perm | Mar/10 | 10 | Apr/11 | 11 |  | Aug/09 | 11 | Feb/10 | 11 |
|  | Isol / Temp | Mar/10 | 10 | Apr/11 | 10 |  | Aug/09 | 10 | Feb/10 | 8 |
| Carajás | Isol / Perm | Apr/12 | 5 | Apr/13 | 5 |  | Nov/11 | 5 | Nov/12 | 5 |
|  | Isol / Temp | Apr/12 | 17 | Apr/13 | 18 |  | Nov/11 | 17 | Nov/12 | 17 |
