# Supplementary material for: Correlates of Zooplankton Beta Diversity in Tropical Lake Systems
Source: PLoS One. 2014 Oct 16;9(10):e109581. doi: 10.1371/journal.pone.0109581 (PMC4199600; doi:10.1371/journal.pone.0109581)
Supplement: Table S2 — Species list for each region. List of species per region and lake category. Dominant species are highlighted in yellow. Tr, Pr, Ar, Ma and Ca = Trombetas, Paraná, Araguaia, Macaé and Carajás, respectively. PC, TI, PI = permanent connected, temporary isolated, permanent isolated. (DOCX) [file pone.0109581.s009.docx]

**Table S2. Species list for each region.** List of species per region and lake category. Dominant species are highlighted in yellow. Tr, Pr, Ar, Ma and Ca = Trombetas, Paraná, Araguaia, Macaé and Carajás, respectively. PC, TI, PI = permanent connected, temporary isolated, permanent isolated.

|  | **Tr-PC** | **Pr-PC** | **Ar-PC** | **Ma-TI** | **Ma-PI** | **Ca-TI** | **Ca-PI** |
| --- | --- | --- | --- | --- | --- | --- | --- |
| **ROTIFERA** |  |  |  |  |  |  |  |
| *Anuraeopsis fissa* | x |  | x | x | x |  |  |
| *Anuraeopsis navicula* |  |  | x |  |  |  | x |
| *Anuraeopsis* sp. | x |  |  |  |  |  |  |
| *Ascomorpha agilis* | x |  |  |  |  |  |  |
| *Ascomorpha ecaudis* | x | x |  | x | x | x | x |
| *Ascomorpha ovalis* |  | x |  |  |  |  |  |
| *Ascomorpha saltans* | x | x |  | x | x |  |  |
| *Asplanchnopus* sp. |  |  |  | x |  | x |  |
| *Asplancna sieboldi* | x | x |  |  |  |  |  |
| *Asplancna* sp. |  |  |  |  | x |  |  |
| *Beauchampiella eudactylota* |  | x | x |  | x | x |  |
| *Brachionus angularis* | x | x | x |  | x |  |  |
| *Brachionus bidentata* |  | x | x |  | x |  |  |
| *Brachionus budapestinensis* |  | x |  |  |  |  |  |
| *Brachionus calyciflorus* |  | x | x |  | x |  |  |
| *Brachionus caudatus* |  | x | x |  | x |  |  |
| *Brachionus dimidiatus* |  |  |  |  |  |  | x |
| *Brachionus dolabratus* | x | x | x |  |  |  |  |
| *Brachionus falcatus* | x | x | x | x | x |  |  |
| *Brachionus forficula* |  | x |  |  |  |  |  |
| *Brachionus gillardi* | x |  |  |  |  |  |  |
| *Brachionus leydigi* | x |  |  |  |  |  |  |
| *Brachionus mirus* | x | x | x |  |  |  |  |
| *Brachionus plicatilis* | x |  |  | x | x |  |  |
| *Brachionus quadridentatus* | x | x | x | x |  |  |  |
| *Brachionus urceolaris* | x |  |  |  |  |  |  |
| *Brachionus zahniseri* | x |  | x |  |  |  |  |
| *Cephalodella gibba* |  | x |  | x | x | x |  |
| *Cephalodella gracilis* |  | x |  |  |  |  |  |
| *Cephalodella hiulca* |  | x |  |  |  | x |  |
| *Cephalodella mucronata* | x | x |  | x |  | x |  |
| *Cephalodella sp* |  |  |  | x | x |  |  |
| *Cephalodella tenuiseta* | x | x |  |  |  |  |  |
| *Collotheca* sp.1 | x |  |  |  |  | x | x |
| *Collotheca* sp.2 |  |  |  |  | x |  |  |
| *Collotheca* sp.3 |  |  |  | x | x |  |  |
| *Colurella obtusa* |  |  | x |  |  |  |  |
| *Colurella* sp. |  |  |  | x | x | x |  |
| *Conochilus coenobasis* |  | x | x |  |  |  |  |
| *Conochilus dossuaris* |  | x |  |  |  |  |  |
| *Conochilus natans* |  | x |  |  |  |  |  |
| *Conochilus* sp. | x |  |  | x | x |  |  |
| *Conochilus unicornis* |  | x |  |  |  |  |  |
| *Dicranophoroides caudatus* |  | x |  |  |  |  |  |
| *Dicranophorus* sp. | x |  |  | x | x | x | x |
| *Dicronophorus forcipatus* |  | x |  |  |  |  |  |
| *Dipleuchlanis propatula* | x | x | x | x | x | x | x |
| *Dissotrocha aculeata* | x |  |  | x |  | x |  |
| *Dissotrocha* sp. |  | x |  |  |  |  |  |
| *Encentrum flexilis* |  |  | x |  |  |  |  |
| *Encentrum* sp. |  |  | x | x | x |  |  |
| *Eothina elongata* |  | x |  |  |  |  |  |
| *Epiphanes brachionus* |  |  | x |  |  |  |  |
| *Epiphanes clavulata* |  | x | x |  |  |  |  |
| *Epiphanes macrourus* |  | x |  |  |  |  |  |
| *Epiphanes* sp. |  | x |  |  |  |  |  |
| *Euchlanis callysta* | x |  |  |  |  |  |  |
| *Euchlanis dilatata* | x | x | x | x | x | x |  |
| *Euchlanis incisa* |  | x |  |  |  |  |  |
| *Euchlanis meneta* |  |  | x |  |  |  |  |
| *Euchlanis triquetra* | x |  |  |  | x |  |  |
| *Filinia longiseta* | x | x | x | x | x |  |  |
| *Filinia opoliensis* | x | x | x |  |  |  |  |
| *Filinia pjeleri* |  | x | x |  |  |  |  |
| *Filinia saltator* |  | x |  |  |  |  |  |
| *Filinia terminalis* | x | x |  |  |  |  |  |
| *Floscularia* sp. |  | x |  |  |  |  |  |
| *Gastropus hiptopus* |  | x |  |  |  |  |  |
| *Gastropus minor* |  |  |  | x | x |  |  |
| *Gastropus* sp. | x | x |  |  |  | x | x |
| *Gastropus stilifer* |  | x |  |  |  |  |  |
| *Hexarthra fennica* |  |  | x |  |  |  |  |
| *Hexarthra intermedia* |  | x |  |  |  |  |  |
| *Hexarthra mira* | x | x | x |  |  |  |  |
| *Hexarthra* sp. |  |  |  | x | x |  |  |
| *Horaella brehmi* |  | x |  |  |  |  |  |
| *Horaella thomassoni* |  | x |  |  |  |  |  |
| *Kellicottia bostoniensis* |  | x |  |  |  |  |  |
| *Keratella americana* | x | x | x |  |  |  |  |
| *Keratella cochlearis* | x | x | x |  | x | x |  |
| *Keratella lenzi* | x | x | x |  | x |  |  |
| *Keratella tropica* |  | x | x |  | x |  |  |
| *Lecane aculeata* |  |  |  | x |  |  |  |
| *Lecane amazonica* |  | x |  |  |  |  |  |
| *Lecane arcuata* |  |  |  |  | x |  |  |
| *Lecane arcula* |  |  |  | x | x |  |  |
| *Lecane boettgeri* |  |  |  | x | x |  |  |
| *Lecane bulla* | x | x | x | x | x | x | x |
| *Lecane closterocerca* | x | x |  | x | x | x |  |
| *Lecane cornuta* | x | x | x | x | x | x |  |
| *Lecane crepida* | x |  |  | x | x |  |  |
| *Lecane curvicornis* | x | x | x | x | x | x |  |
| *Lecane deridderae* |  |  |  | x | x | x |  |
| *Lecane doryssa* |  |  |  | x | x |  |  |
| *Lecane dumonti* |  |  |  | x |  | x |  |
| *Lecane elegans* | x |  |  |  | x |  |  |
| *Lecane elsa* |  | x | x |  |  |  |  |
| *Lecane eutarsa* | x |  |  | x | x | x |  |
| *Lecane flexilis* | x |  |  |  |  |  |  |
| *Lecane furcata* | x | x | x | x | x | x |  |
| *Lecane grandis* |  |  |  | x | x |  |  |
| *Lecane haliclysta* |  | x | x | x | x | x |  |
| *Lecane hamata* | x | x |  | x | x | x |  |
| *Lecane hornemanni* | x |  |  | x | x | x |  |
| *Lecane imbricata* |  |  | x |  |  |  |  |
| *Lecane inermis* |  |  |  | x | x |  |  |
| *Lecane inopinata* | x | x |  |  |  |  |  |
| *Lecane leontina* | x | x | x | x | x | x | x |
| *Lecane ludwigii* | x | x | x | x | x | x | x |
| *Lecane luna* |  | x | x |  |  |  |  |
| *Lecane lunaris* | x | x | x | x | x | x | x |
| *Lecane melini* | x |  | x |  |  |  |  |
| *Lecane monostyla* | x | x | x |  | x |  |  |
| *Lecane nana* |  |  |  |  | x |  |  |
| *Lecane obtusa* |  |  |  | x | x | x |  |
| *Lecane ovalis* |  | x |  |  |  |  |  |
| *Lecane papuana* |  | x | x | x | x |  |  |
| *Lecane pertica* | x | x |  |  |  |  |  |
| *Lecane proiecta* | x | x | x |  |  |  |  |
| *Lecane punctata* |  |  |  | x | x | x |  |
| *Lecane pusilla* | x | x |  |  | x |  |  |
| *Lecane pyriformis* |  |  |  | x | x | x |  |
| *Lecane quadridentata* | x | x | x | x | x | x | x |
| *Lecane rhenana* | x |  |  |  |  | x |  |
| *Lecane rhytida* |  | x |  | x | x | x |  |
| *Lecane robertsonae* | x |  |  |  |  |  |  |
| *Lecane rudescui* | x |  |  |  |  |  |  |
| *Lecane scutata* |  |  |  | x | x | x | x |
| *Lecane signifera* | x | x |  | x | x | x | x |
| *Lecane* sp.1 |  |  | x | x | x |  |  |
| *Lecane spinulifera* |  |  |  |  | x |  |  |
| *Lecane stenroosi* |  |  |  | x | x |  |  |
| *Lecane stichaea* | x | x | x | x | x | x |  |
| *Lecane stichoclysta* | x |  |  | x | x | x |  |
| *Lecane subtilis* | x |  |  | x | x | x |  |
| *Lecane thalera* |  |  |  | x | x |  |  |
| *Lecane uenoi* | x |  |  |  |  |  |  |
| *Lecane undulata* | x |  |  |  | x |  |  |
| *Lecane ungulata* |  |  | x |  |  | x |  |
| *Lepadella benjamini* |  | x |  |  |  |  |  |
| *Lepadella dactylicta* | x |  | x |  |  |  |  |
| *Lepadella imbricata* |  | x |  |  |  |  |  |
| *Lepadella patella* | x | x | x | x | x | x |  |
| *Lepadella quinquecostata* | x |  |  | x |  |  |  |
| *Lophocharis oxysternon* |  | x |  |  |  |  |  |
| *Lophocharis salpina* |  | x | x |  |  |  |  |
| *Lophocharis* sp. |  |  |  |  | x |  |  |
| *Macrochaetus altamirai* | x |  |  |  |  | x |  |
| *Macrochaetus collinsi* | x | x | x | x | x | x | x |
| *Macrochaetus kostei* | x |  |  | x | x | x |  |
| *Macrochaetus longipes* | x |  |  |  |  |  |  |
| *Macrochaetus sericus* |  | x | x |  |  | x | x |
| *Monommata actices* |  | x |  |  |  |  |  |
| *Monommata dentata* |  | x |  |  |  |  |  |
| *Monommata maculata* |  | x |  |  |  |  |  |
| *Monommata* sp. | x | x |  | x | x | x |  |
| *Mytilina acanthophora* |  | x | x |  |  | x |  |
| *Mytilina bissulcata* | x | x | x | x | x |  |  |
| *Mytilina macrocera* | x | x | x |  |  |  |  |
| *Mytilina mucronata* | x | x | x |  |  |  |  |
| *Mytilina ventralis* | x | x | x |  |  |  |  |
| *Notholca* sp. | x |  |  |  |  | x |  |
| *Notommata copeus* |  | x |  | x | x | x |  |
| *Notommata pachyura* |  | x | x |  |  |  |  |
| *Notommata saccigera* |  |  | x |  |  |  |  |
| *Notommata* sp. | x |  |  | x | x | x |  |
| *Paracolurella logima* |  |  |  |  |  | x |  |
| *Plationus macrachantus* |  | x |  |  |  |  |  |
| *Plationus patulus* | x | x | x | x |  | x |  |
| *Platyas leloupi* | x | x | x | x | x | x |  |
| *Platyas quadricornis* |  | x | x |  |  |  |  |
| *Ploesoma* sp. |  |  |  | x | x |  |  |
| *Ploesoma truncatum* | x | x |  |  |  |  |  |
| *Polyarthra dolichoptera* | x | x | x | x | x | x | x |
| *Polyarthra vulgaris* |  | x | x |  |  |  |  |
| *Pompholyx complanata* |  | x |  |  |  |  |  |
| *Pompholyx triloba* |  | x |  |  |  |  |  |
| *Ptygura libera* | x |  |  |  |  | x | x |
| *Ptygura* sp. |  | x | x |  |  |  |  |
| *Scaridium longicaudum* |  |  |  | x | x |  |  |
| *Sinantherina* sp. | x |  |  |  |  |  |  |
| *Squatinella leydigi* |  |  |  |  |  | x |  |
| *Squatinella longispinata* |  |  |  | x |  |  |  |
| *Squatinella mutica* |  |  | x |  |  |  |  |
| *Squatinella rostrum* |  |  |  | x | x | x |  |
| *Stenophorus fimbriatus* | x |  |  |  |  |  |  |
| *Synchaeta oblonga* |  | x |  |  |  |  |  |
| *Synchaeta pectinata* |  | x |  |  |  |  |  |
| *Synchaeta* sp. | x |  |  | x | x | x | x |
| *Synchaeta stylata* |  | x | x |  |  |  |  |
| *Testudinella discoidea* |  | x |  |  |  |  |  |
| *Testudinella mucronata* | x | x |  | x | x |  |  |
| *Testudinella ohlei* | x | x |  | x | x | x |  |
| *Testudinella patina* | x | x | x | x | x | x | x |
| *Testudinella patina dendradena* |  | x |  |  |  |  |  |
| *Testudinella tridentata* | x |  |  |  |  |  |  |
| *Trichocerca bicristata* | x | x | x | x | x | x |  |
| *Trichocerca bidens* | x | x | x | x | x | x |  |
| *Trichocerca capucina* | x | x |  |  |  | x | x |
| *Trichocerca chatonni* | x | x |  |  |  |  |  |
| *Trichocerca collaris* | x |  |  | x | x | x |  |
| *Trichocerca cylindrica* | x | x | x |  |  | x |  |
| *Trichocerca dixonnuttalli* |  | x |  |  |  |  |  |
| *Trichocerca elongata* |  | x | x |  |  |  |  |
| *Trichocerca fusiformis* |  |  | x |  |  |  |  |
| *Trichocerca gracillis* |  | x | x |  |  |  |  |
| *Trichocerca heterodactyla* |  | x |  |  |  |  |  |
| *Trichocerca iernis* |  | x | x | x | x | x |  |
| *Trichocerca inermis* |  | x | x | x | x |  |  |
| *Trichocerca insignis* | x | x |  | x | x | x | x |
| *Trichocerca insulana* |  | x | x |  |  |  |  |
| *Trichocerca longiseta* |  | x |  |  |  |  |  |
| *Trichocerca montana* | x |  |  | x | x | x |  |
| *Trichocerca myersi* |  | x |  |  |  |  |  |
| *Trichocerca porcellus* | x |  |  | x | x |  |  |
| *Trichocerca pusilla* | x | x | x | x | x | x | x |
| *Trichocerca rousseleti* |  | x |  |  |  |  |  |
| *Trichocerca ruttneri* |  | x |  |  |  |  |  |
| *Trichocerca scipio* |  | x |  | x | x | x |  |
| *Trichocerca similis similis* | x | x | x | x | x | x | x |
| *Trichocerca stylata* |  | x |  |  |  |  |  |
| *Trichocerca tenuidens* | x |  |  |  |  |  |  |
| *Trichocerca tigris* |  | x | x |  |  |  |  |
| *Trichocerca tridentata* |  | x |  |  |  |  |  |
| *Trichotria tetractis* | x | x | x |  |  | x |  |
| *Trochosphaera* sp. | x |  |  |  |  |  |  |
| **Number of rotifer species** | **104** | **136** | **83** | **86** | **96** | **72** | **24** |
| **CLADOCERA** |  |  |  |  |  |  |  |
| *Acantholeberis* sp. |  |  |  | x |  |  |  |
| *Acroperus tupinamba* | x | x |  | x | x |  |  |
| *Alona dadivi* | x |  | x | x | x | x |  |
| *Alona dentifera* | x | x | x | x | x | x |  |
| *Alona glabra* | x |  | x |  |  | x |  |
| *Alona guttata* | x | x | x | x |  | x |  |
| *Alona iheringula* | x |  | x | x | x | x |  |
| *Alona intermedia* | x | x |  | x | x | x | x |
| *Alona ossiani* | x | x | x | x | x | x | x |
| *Alona* sp. |  | x |  |  |  |  |  |
| *Alonella clathracula* | x |  |  | x | x | x |  |
| *Alonella dadayi* | x | x | x | x | x | x | x |
| *Alonella* sp. | x | x |  |  |  |  |  |
| *Anthalona verrucosa* | x | x |  | x | x | x | x |
| *Bosmina frey* |  | x |  |  |  |  |  |
| *Bosmina hagmanni* | x | x | x | x | x |  |  |
| *Bosmina longirostris* |  |  | x |  |  |  |  |
| *Bosmina tubicen* |  | x | x |  |  |  |  |
| *Bosminopsis brandorfii* | x |  |  |  |  |  |  |
| *Bosminopsis deitersi* | x | x | x | x | x |  | x |
| *Bosminopsis negrensis* | x |  |  |  |  |  |  |
| *Camptocercus australis* |  | x |  | x | x |  |  |
| *Ceriodaphnia cornuta* | x | x | x | x | x | x | x |
| *Ceriodaphnia laticaudata* |  | x |  |  |  |  |  |
| *Ceriodaphnia quadrangula* |  |  | x |  |  |  |  |
| *Ceriodaphnia silvestrii* |  | x | x |  |  |  |  |
| *Ceriodaphnia* sp. |  | x |  |  |  |  |  |
| *Chydorus eurynotus* | x | x | x | x | x |  | x |
| *Chydorus parvareticulatus* |  | x |  |  |  |  |  |
| *Chydorus pubescens* | x | x | x | x | x | x | x |
| *Chydorus sphaericus* |  | x | x |  |  |  |  |
| *Coronatella monacantha* |  | x | x | x | x |  |  |
| *Coronatella poppei* |  | x | x |  |  |  |  |
| *Coronatella rectangula* |  | x |  |  |  |  |  |
| *Dadaya macrops* |  |  |  | x | x |  |  |
| *Daphnia gessneri* |  | x | x |  |  |  |  |
| *Daphnia laevis* |  | x | x |  |  |  |  |
| *Diaphanosoma birgei* | x | x | x | x | x | x | x |
| *Diaphanosoma brevireme* |  | x |  |  |  |  |  |
| *Diaphanosoma fluviatile* |  | x | x |  |  |  |  |
| *Diaphanosoma polyspina* | x |  |  |  |  |  |  |
| *Diaphanosoma* sp. | x |  |  |  |  |  |  |
| *Diaphanosoma spinulosum* |  | x |  |  |  |  |  |
| *Disparalona hamata* |  | x | x |  |  |  |  |
| *Disparalona leptorhyncha* | x |  |  | x | x | x |  |
| *Dunhevedia americana* |  |  |  | x | x |  |  |
| *Dunhevedia odontoplax* |  |  |  | x | x | x |  |
| *Ephemeroporus barroisi* | x |  | x | x | x | x | x |
| *Ephemeroporus hybridus* |  | x |  | x | x | x |  |
| *Ephemeroporus tridentatus* |  | x |  | x | x |  |  |
| *Euryalona brasiliensis* |  | x | x |  |  |  |  |
| *Euryalona orientalis* |  | x | x |  |  |  |  |
| *Graptoleberisoccidentalis* | x | x |  | x | x | x |  |
| *Grimaldina brazzai* | x | x |  | x |  | x | x |
| *Guernella raphaellis* |  | x |  | x | x |  |  |
| *Holopedium amazonicum* | x |  |  |  |  |  |  |
| *Ilyocryptus spinifer* | x | x | x | x | x | x | x |
| *Karualona muelleri* | x |  | x | x | x | x |  |
| *Kurzia polyspina* | x | x |  | x | x | x |  |
| *Latonopsis australis* |  |  |  | x | x | x |  |
| *Leydigia propinqua* |  |  |  | x | x |  |  |
| *Leydigiopsis brevirostris* |  |  | x | x | x |  |  |
| *Leydigiopsis curvirostris* |  |  |  |  |  | x | x |
| *Leydigiopsis megalops* | x |  | x |  |  |  |  |
| *Macrothrix elegans* |  | x |  | x | x | x | x |
| *Macrothrix laticornis* | x |  |  | x | x | x |  |
| *Macrothrix mira* | x |  |  |  |  |  |  |
| *Macrothrix paulensis* | x |  |  | x | x |  |  |
| *Macrothrix sioli* | x |  |  |  |  | x |  |
| *Macrothrix spinosa* |  |  | x |  |  |  | x |
| *Macrothrix squamosa* |  | x |  |  |  |  |  |
| *Macrothrix superaculeata* | x |  |  |  |  |  |  |
| *Moina micrura* |  | x | x | x | x |  |  |
| *Moina minuta* | x | x | x |  |  |  |  |
| *Moina reticulata* |  | x |  |  |  |  |  |
| *Moinodaphnia macleayi* |  |  |  | x | x |  |  |
| *Nicsmirnovius fitzpatricki* |  | x |  |  |  |  |  |
| *Nicsmirnovius* sp. | x |  |  |  |  |  |  |
| *Notoalona sculpta* | x | x |  | x | x | x |  |
| *Oxyurella longicaudis* |  |  | x | x | x |  |  |
| *Parvalona parva* | x |  |  |  |  | x |  |
| *Pleuroxus similis* |  |  | x |  |  |  |  |
| *Pseudosida ramosa* | x | x |  | x | x | x | x |
| *Scapholeberis armata* |  |  |  | x | x |  |  |
| *Simocephalus strictum* |  | x |  | x | x |  |  |
| *Simocephalus vetulus* | x |  |  | x |  |  |  |
| *Streblocerus pygmaeus* | x |  |  | x |  | x |  |
| **Number of cladoceran species** | **43** | **50** | **36** | **47** | **42** | **31** | **16** |
| **COPEPODA** |  |  |  |  |  |  |  |
| *Apocyclops* sp. |  |  |  |  | x |  |  |
| *Argyrodiaptomus azevedoi* |  | x |  |  |  |  |  |
| *Argyrodiaptomus paggi* |  |  |  |  |  | x |  |
| *Aspinus acicularis* | x |  |  |  |  |  |  |
| *Diptomus azureus* |  |  |  | x | x |  |  |
| *Ectocyclops rubescens* |  |  |  | x | x |  |  |
| *Eucyclops* sp. |  |  |  |  |  | x |  |
| *Halicyclops* sp. |  |  |  | x | x |  |  |
| *Mesocyclops aspericornis* |  | x |  |  |  |  |  |
| *Mesocyclops ellipticus* |  |  | x |  |  |  |  |
| *Mesocyclops longisetus* | x | x | x | x | x | x | x |
| *Mesocyclops meridianus* |  | x |  |  |  |  |  |
| *Mesocyclops ogunus* |  | x |  |  |  |  |  |
| *Mesocyclops* sp. | x | x | x |  |  |  |  |
| *Metacyclops laticornis* |  | x |  |  |  |  |  |
| *Metacyclops mendocinus* |  | x |  |  |  |  |  |
| *Microcyclops alius* |  |  |  | x |  |  | x |
| *Microcyclops anceps* |  | x | x | x | x | x | x |
| *Microcyclops ceibaensis* | x |  |  | x | x |  |  |
| *Microcyclops* sp. | x | x |  |  |  |  |  |
| *Microcylops finitimus* |  |  |  |  | x | x |  |
| *Notodiaptomus amazonicus* |  | x | x |  |  |  |  |
| *Notodiaptomus cearensis* |  |  |  | x | x |  |  |
| *Notodiaptomus coniferoides* | x |  |  |  |  |  |  |
| *Notodiaptomus dahli* |  |  | x |  |  |  |  |
| *Notodiaptomus deitersi* |  | x |  |  |  |  | x |
| *Notodiaptomus henseni* |  | x |  |  |  |  |  |
| *Notodiaptomus iheringi* |  | x |  |  |  |  |  |
| *Notodiaptomus isabelae* |  | x |  |  |  |  |  |
| *Notodiaptomus kieferi* |  | x |  |  |  |  |  |
| *Notodiaptomus maracaibensis* |  |  | x |  |  |  |  |
| *Notodiaptomus paraensis* |  |  |  |  |  | x |  |
| *Notodiaptomus* sp. |  | x |  |  |  |  |  |
| *Oithona amazonica* | x |  |  |  |  |  |  |
| *Paracyclops chiltoni* |  | x |  |  |  |  |  |
| *Paracyclops fimbriatus* |  | x |  |  | x |  |  |
| *Paracyclops* sp. |  | x |  |  |  | x |  |
| *Pseudodiaptomus richardi* |  |  |  |  | x |  |  |
| *Rhacodiaptomus besti* | x |  |  |  |  |  |  |
| *Rhacodiaptomus retroflexus* | x |  |  |  |  |  |  |
| *Termocyclops decipiens* |  | x | x |  |  |  |  |
| *Thermocyclops minutus* |  | x | x |  |  |  |  |
| *Thermocyclops* sp. |  |  |  |  | x |  |  |
| *Tropocyclops federensis* |  |  |  | x |  |  |  |
| *Tropocyclops nananae* |  |  |  | x | x | x | x |
| *Tropocyclops prasinus* |  |  |  | x | x | x | x |
| Anostraca |  |  |  |  |  | x |  |
| **Number of copepod species** | **9** | **22** | **9** | **11** | **14** | **10** | **6** |
| **Total number of species** | **156** | **208** | **128** | **144** | **152** | **113** | **46** |
