# Supplementary material for: Correlates of Zooplankton Beta Diversity in Tropical Lake Systems
Source: PLoS One. 2014 Oct 16;9(10):e109581. doi: 10.1371/journal.pone.0109581 (PMC4199600; doi:10.1371/journal.pone.0109581)
Supplement: Table S4 — Linear mixed-effects models of zooplankton beta diversity measured as the mean Bray-Curtis and Simpson distances to group centroid for disturbance data. Summary of the linear mixed-effects models of zooplankton beta diversity measured as the mean Bray Curtis (DistCBC) and Simpson (DistCSim) distance to group centroid for disturbance data (permanent and temporary aquatic systems from Macaé and Carajás). Marginal R2 represents the variance explained by fixed factors. (DOCX) [file pone.0109581.s011.docx]

**Table S4. Linear mixed-effects models of zooplankton beta diversity measured as the mean Bray-Curtis and Simpson distances to group centroid for disturbance data.** Summary of the linear mixed-effects models of zooplankton beta diversity measured as the mean Bray Curtis (DistC_BC_) and Simpson (DistC_Sim_) distance to group centroid for disturbance data (permanent and temporary aquatic systems from Macaé and Carajás). Marginal R^2^ represents the variance explained by fixed factors.

| Samples sizes: n = 16 observations | | | | | | |
| --- | --- | --- | --- | --- | --- | --- |
| Group: regions = 2 | | | | | | |
| Marginal R^2^ = 0.70 | | | | | | |
| DistC_BC_ | Random effect | Variance component | | | | |
|  | Region | 0.0017 |  |  |  |  |
|  | Residual | 0.0008 |  |  |  |  |
|  | Fixed effects | Estimate | SE | *df* | *t* | *P* |
|  | Intercept | 0.500 | 0.042 |  | 11.833 |  |
|  | Disturbance (temp) | -0.082 | 0.022 | 1 | -3.713 | 0.006 |
|  | Environmental heterogeneity | 0.012 | 0.016 | 1 | 0.738 | 0.54 |
|  | Spatial extent | 0.007 | 0.231 | 1 | 0.032 | 0.97 |
|  | Seasonality (wet) | -0.005 | 0.015 | 1 | -0.317 | 0.76 |
| Marginal R^2^ = 0.80 | | | | | | |
| DistC_Sim_ | Random effect | Variance component | | | | |
|  | Region | 0.0005 |  |  |  |  |
|  | Residual | 0.0013 |  |  |  |  |
|  | Fixed effects | Estimate | SE | *df* | *t* | *P* |
|  | Intercept | 0.413 | 0.138 |  | 2.987 |  |
|  | Disturbance (temp) | -0.118 | 0.021 | 1 | -5.610 | 0.0005 |
|  | Environmental heterogeneity | 0.029 | 0.018 | 1 | 1.590 | 0.33 |
|  | Spatial extent | -0.275 | 0.874 | 1 | -0.315 | 0.62 |
|  | Seasonality (wet) | 0.023 | 0.019 | 1 | 1.245 | 0.26 |
