# Supplementary material for: Correlates of Zooplankton Beta Diversity in Tropical Lake Systems
Source: PLoS One. 2014 Oct 16;9(10):e109581. doi: 10.1371/journal.pone.0109581 (PMC4199600; doi:10.1371/journal.pone.0109581)
Supplement: Table S5 — Linear mixed-effects models of environmental heterogeneity for connectivity and disturbance datasets. Summary of the linear mixed-effects models of environmental heterogeneity (Env) measured as the mean Euclidean distance to group centroid for connectivity (connected and isolated permanent lakes from all studied regions) and disturbance datasets (permanent and temporary aquatic systems from Macaé and Carajás). Marginal R2 represents the variance explained by fixed factors. (DOCX) [file pone.0109581.s012.docx]

**Table S5. Linear mixed-effects models of environmental heterogeneity for connectivity and disturbance datasets.** Summary of the linear mixed-effects models of environmental heterogeneity (Env) measured as the mean Euclidean distance to group centroid for connectivity (connected and isolated permanent lakes from all studied regions) and disturbance datasets (permanent and temporary aquatic systems from Macaé and Carajás). Marginal R^2^ represents the variance explained by fixed factors.

| Samples sizes: n = 18 observations | | | | | | |
| --- | --- | --- | --- | --- | --- | --- |
| Group: regions = 5 | | | | | | |
| Marginal R^2^ = 0.47 | | | | | | |
| Env | Random effect | Variance component | | | | |
|  | Region | 0.6061 |  |  |  |  |
|  | Residual | 0.1009 |  |  |  |  |
|  | Fixed effects | Estimate | SE | *df* | *t* | *P* |
|  | Intercept | 0.490 | 0.865 |  | 0.566 |  |
|  | Connectivity (isolated) | 1.459 | 0.863 | 1 | 1.691 | 0.080 |
|  | Spatial extent | -0.467 | 1.985 | 1 | -0.236 | 0.834 |
|  | Seasonality (wet) | 0.068 | 0.149 | 1 | 0.455 | 0.657 |
| Samples sizes: n = 16 observations | | | | | | |
| Group: regions = 2 | | | | | | |
| Marginal R^2^ = 0.27 | | | | | | |
| Env | Random effect | Variance component | | | | |
|  | Region | 0.7251 |  |  |  |  |
|  | Residual | 0.3361 |  |  |  |  |
|  | Fixed effects | Estimate | SE | *df* | *t* | *P* |
|  | Intercept | 1.768 | 0.714 |  | 2.477 |  |
|  | Disturbance (temporary) | -0.372 | 0.332 | 1 | -1.120 | 0.29 |
|  | Spatial extent | 5.557 | 3.912 | 1 | 1.421 | 0.18 |
|  | Seasonality (wet) | -0.435 | 0.289 | 1 | -1.504 | 0.16 |
